# Romanian medical research in the context of European integration

**Published:** 2008-04-15

**Authors:** Popa Florian

**Affiliations:** * Rector of ”Carol Davila” University of Medicine and Pharmacy, Bucharest, Romania

**Figure:**
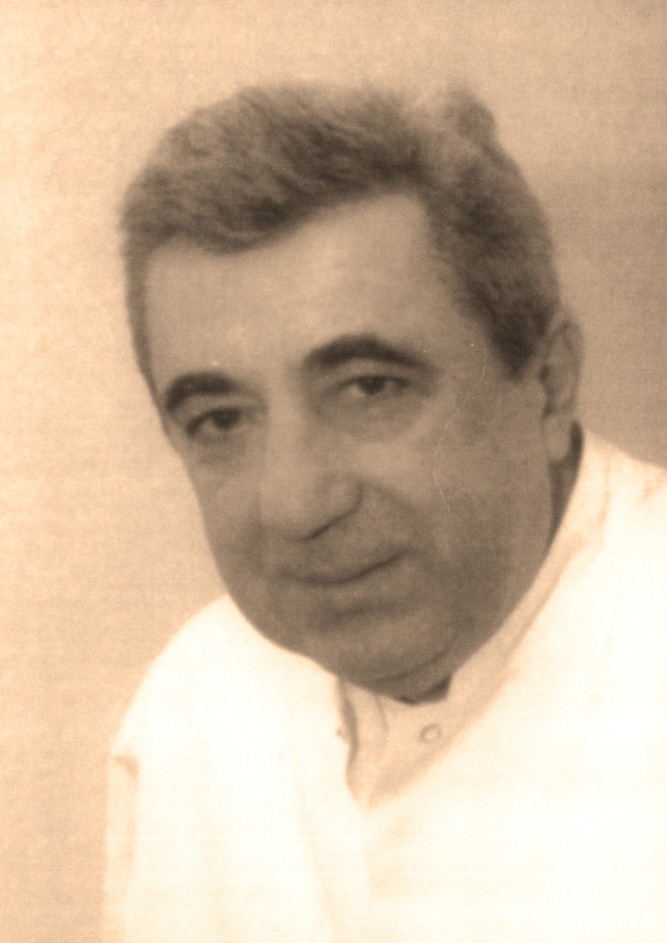


Romanian medical research – a field that was ostracized before and many years after the Revolution – is beginning to aquire its own identity within the European context.

In comparison with the clinical practice, medical research has known a long period of stagnation. It was almost entirely abandoned due to the lack of funds.

Medicine expects from research solutions for the problems which it can not find answers. 

One of the features of Romanian research is the fact that, at the institutional level of elaboration of a development strategy and policy and also of the research activity assertion, it is not regarded as an economical power like in the case of strong economic countries, but merely as a domain of job conservation. Thus, a minimal existence budget is given to it, which can be obtained through a competition based, in general, on criteria far from European standards.

As a consequence, a difficulty in establishing priority directions of research related to the requirements of economic progress arises on one hand and a difficulty in imposing real competition criteria on another hand.

Another feature of Romanian medical research, especially of fundamental research, which at European level is achieved in 80% of cases in higher education institutions, is the lack of research performance criteria used for getting scientific and university titles. Unlike the situation in Romania, in occidental medicine, the academic promotion is done mainly on research results.

It is difficult to appreciate which of the two branches of research is the most important: fundamental or applicable research. One thing is for sure: a balance between the two must be realized because without fundamental research, research which generates knowledge, no applicable research will exist for long.

If fundamental research can develop in higher education institutions predominantly and is financed by budget resources, applicable research should take place in national research institutions and attract private investment funds as it is practiced for the scientific research policy in Europe or in the world’s powerful economies.

At present, there is a rather dissipated research in Romania, by tackling too many fields in an unorganized fashion, laboratories function independently of each other and most of the times the results of Romanian research come to be known by Romanian researchers only after publication in international journals, a fact that is not appropriate. A policy of concentrating laboratories and avoid their overlap should be elaborated.

Healthcare research should represent a national priority in accordance with the necessities and priorities specific to our country in European geography, being based on a strategic plan of medical research which takes into account the necessity of modern technological approach for both pathological and physiological logic of the living.

As a consequence, priority fields of research must be outlined, towards which most of the budget is to be distributed, a real competition among projects must exist and be based on correct and responsible evaluation with credible and internationally accredited criteria and there must be projects that generate either knowledge or results that are able to be put into practice.

A great attention and a particular institutional responsibility is required, especially for fundamental research, in approaching the process of making an effective relation between higher education and research activity carried on in universities, for their mutual advantage.

For a country known for its special potential of intelligence, Romania is a place where too few original research projects or projects capable to find room in international prestigious journals are carried out. There are multiple explanations, the majority having financial substratum like difficulties in obtaining funds for great span projects, low number of grants available in Romania and the birocracy that discourages many candidates, which anyhow is less tough and less detailed than in the European Union.

The European integration implies also the opportunity for Romanian medical research, at least for the next 10 years, to be able to actively take part in projects of bioengineering, biotechnology and nano medicine, which prefigures the future of medicine.

Progress in medicine is impossible without the investment in research. Wide access to therapy is impossible without low cost drugs. Hence, the path must be opened by original products and, after patent expiration, generic drugs will have the role of enabling the access of a greater number of patients to therapies. However, the future does not belong neither to generics, not to brands but to the reasonable balance between the two.

Past years have brought some investments in the general field of research but access to these funds, as well as the access to the European funds, a perspective which becomes real today, depends essentially on the qualification of specialists, groups capable of working under performance conditions and standards equal to those of other EU countries, as well as on their desire to apply their experience in this country and establish local centers that affiliate to more powerful and maybe more technologically advanced centers in other European countries

Also, there is a necessity for real projects to access these funds, projects which need to prove their importance for the evolution of medicine and lead towards solutions in disease management for the medicine of the future.

Such a project belongs to a research staff from the Fundeni General Surgery and Liver Transplant Clinic, who have succeeded to undertake the research for pancreatic and colon cancer, trying to identify the genetic determinants of these diseases.

The unfolding of research activity within the research departments in clinical hospitals, although regarded by many a luxury that the health system should not afford, represents in fact a prerequisite for the progress of Romanian medicine.

The excellence research program as well as the considerable financial resources of the FP7 program which can also be accessed by research departments within clinical hospitals, enables Romanian medical research to presently benefit from a substantial material and financial support.

In this context, the department of research in neurosciences of Bagdasar-Arseni Emergency Clinical Hospital has achieved important advances in the development of fundamental medical research, being involved in far-reaching projects.

The main research field is represented by the study of cerebral tumors biology, especially of those tumors with high malignancy. Among these, a particular interest is raised by the glioblastoma, due to its remarkably severe prognosis and inefficiency of present therapeutical options.

To this effect, a first step of this project was constituted by the creation of a cerebral tumor bank where a number of tumor samples (100 at present) were preserved in liquid nitrogen at a temperature of -180 degrees Celsius, samples which make all of the histopathological spectrum: from high malignant tumors (glioblastoma, medulloblastoma, brain metastases) to rare tumors of the CNS (central neurocytoma, hemangiopericytoma) along with establishing collaboration relationships with various national and international institutions which receive tumoral samples in order to attain complex experiences in the field of molecular and cellular biology.

An important achievement of the research department is the Excellence Research project called “Experimental studies on mesenchymale human stem cells transfected for the purpose of developing an innovative therapeutical management of glioblastoma”, a complex project of fundamental research which benefits from substantial funds and surmises the cooperation with 2 more research institutes and 2 universities. As part of the project, mesenchymale stem cells cultures will be transferred to the hospital’s research department of neurosciences, thus creating the necessary environment for the development of a new path of research, namely neuroregeneration.

The University of Medicine and Pharmacy Carol Davila is also closely involved in the main priority European research trends, which through the “George Emil Palade” program engages in the concept of trans national medicine.

The future of Romanian medical research depends solely on the ability of Romanian specialists to come up with results with the help of the grants which they receive. In this context, Romania, as a European Union member, invests more and more in research, in an organized framework with grants that are ascribed by competition, with results directly ready to be put into practice.
